# Defining Cachexia in Children With Cancer in the United States: Developing a Framework to Inform Clinical and Research Practice

**DOI:** 10.1111/jhn.70325

**Published:** 2026-08-13

**Authors:** Daniel V. Runco, Guohai Zhou, Adina R. Lemeshow, Stephen E. Schachterle, Agnieszka Gajewska, Ira Jacobs, Alexandra Palmer, Xunming Sun

**Affiliations:** ^1^ Division of Hematology, Oncology, Bone Marrow Transplant & Cellular Therapy, Department of Pediatrics, Seattle Children's Hospital University of Washington Seattle Washington USA; ^2^ Ben Towne Center for Childhood Cancer Research Seattle Children's Research Institute Seattle Washington USA; ^3^ Fred Hutchinson Cancer Research Center Seattle Washington USA; ^4^ Global Medical Epidemiology Pfizer Inc New York New York USA; ^5^ Global Medical Epidemiology Pfizer Polska Warsaw Poland; ^6^ Formerly Pfizer Research and Development Pfizer Inc New York New York USA; ^7^ Pfizer Research and Development Pfizer Inc New York New York USA

**Keywords:** cachexia, cancer, children, malnutrition, paediatric, weight loss

## Abstract

**Background:**

Cancer cachexia describes the muscle and weight loss, anorexia, and physical impairment common in certain adult cancers. No formal criteria exist to define paediatric cancer cachexia, making its clinical recognition and impact largely unknown. Using established undernutrition guidelines, criteria were developed to identify potential cachexia in children with cancer based on standardised anthropometric measures or diagnostic codes.

**Methods:**

The Surveillance, Epidemiology, and End Results and Optum Electronic Health Records databases were used to estimate prevalence and incidence rates of cancer and cancer cachexia between 2017 and 2021 in patients aged < 20 and < 18 years, respectively. In the absence of a consensus definition for paediatric cachexia, four sets of candidate criteria were developed based on body weight for length/height or body mass index z‐scores, or reduced gain or loss of body weight, corresponding to mild or severe undernutrition. A fifth criterion required a potentially cachexia‐related ICD‐10‐CM diagnosis code.

**Results:**

This study included 26,855 paediatric patients (aged < 18 years) with cancer; ~90% were aged ≥ 2 years. Epidemiologic estimates for cancer cachexia prevalence and incidence rate varied considerably by age, cancer type and cachexia definition. Estimates were low for most categories, especially in the youngest age groups; however, cachexia was difficult to assess in patients aged < 2 years due to small patient numbers.

**Conclusions:**

We describe one of the first attempts to develop potential paediatric cachexia criteria. These criteria and resulting data provide a framework to inform further research into generating consensus guidelines for the identification of childhood cancer cachexia.

## Summary

- Knowledge around paediatric cancer cachexia is limited; lacking a formal definition or standardised criteria leads to under‐recognition and inconsistent data reporting. This retrospective study proposed five candidate diagnostic criteria, developed to estimate paediatric cancer cachexia prevalence and incidence in the United States.
- While generally lower in paediatric patients with cancer vs adult patients with cancer, cancer cachexia estimates for prevalence and incidence varied by age, cancer type, and definition used, underscoring the complexity of establishing standardised paediatric criteria
- These candidate diagnostic criteria provide a framework for future consensus definitions of paediatric cancer cachexia, which may support improved recognition and management of this condition in children

**Abbreviations**
AML: acute myeloid leukemia
CNS: central nervous system
FDA: United States Food and Drug Administration
ICD‐10‐CM: International Classification of Diseases, Tenth Revision, Clinical Modification
IP: inpatient
IQR: interquartile range
N/A: not available
OP: outpatient
Optum EHR: Optum Enriched Oncology Electronic Health Records Database
PY: person years
QoL: quality of life
SD: standard deviation
SEER: Surveillance, Epidemiology, and End Results

## 1 Introduction

Cachexia is a multifactorial and multi‐organ condition characterised by progressive loss of muscle and body fat, systemic inflammation and significant metabolic abnormalities [1]. This complex disorder manifests through signs and symptoms such as reduced appetite and food intake, involuntary weight loss, changes in body composition, fatigue and worsening physical function [2, 3, 4, 5]. Cancer‐related cachexia can occur in up to 70%–80% of adult patients depending on tumour type and stage, where it is associated with increased treatment‐related morbidity and overall mortality [6, 7, 8, 9]. Furthermore, cachexia also negatively impacts treatment delivery, patient quality of life (QoL), and activities of daily living [10, 11, 12, 13]. The pathophysiology of cachexia involves not only inadequate nutrition as a result of nausea, vomiting and anorexia, but also a combination of systemic inflammation, hormonal imbalances, and disruptions in protein synthesis and breakdown resulting in muscle loss and end organ damage [14, 15]. These factors contribute to the condition's resistance to nutritional interventions alone, necessitating a comprehensive approach to management that addresses the underlying disease and its multifaceted impacts [16]. Despite cachexia remaining a serious and debilitating condition with high unmet medical needs, the negative effects on patients' QoL and clinical outcomes have failed to prompt significant research in paediatric cancer cachexia [17, 18].

International consensus has helped to define cancer cachexia for adults. A standardised, widely accepted set of criteria to describe body weight loss in cachexia has improved the accuracy and consistency of diagnosis [1]. In contrast, there is no formal definition or set of criteria to diagnose paediatric cachexia [17, 18]. Current cachexia definitions and criteria are based on adults, with the assumption that a healthy body is fully developed and should be static in height; however, this does not apply to children, who are continuing to grow and develop. The only clinically available criteria describing the loss of body weight in children are definitions for ‘malnutrition’ or ‘undernutrition’ [19, 20, 21]. However, cachexia, a more complex phenotype beyond weight loss alone, is not interchangeable with malnutrition or undernutrition. In children, undernutrition is best assessed using standardised anthropometric measures normalised to age and biologic sex, including changes from the mean body weight‐for‐height/length, body mass index‐for‐age, and length/height‐for‐age measured in standard distribution units assuming a standard normal distribution (i.e., z‐scores) [20]. Additional indicators can be captured, such as body weight gain velocity, body weight loss and deceleration in growth trajectory [20]. Although malnutrition has been shown to worsen treatment‐related toxicity and overall survival, it fails to account for changes in body composition, impact on physical function, and other physiological changes that may affect drug metabolism, QoL, and tolerance and response to cancer therapy. The impact of childhood cancer cachexia may be even more profound given the potential deleterious effects on long‐term growth; bone maturation and mineral density; motor, cognitive, and neurologic development; and mortality [18, 19, 22, 23].

The lack of criteria for defining paediatric cancer cachexia may result in inadequate clinical recognition and means that its impact is largely unknown [17]. Without a consensus definition, it is challenging to document cancer cachexia conclusively in paediatric patients. The aim of this study was to develop and assess different candidate diagnostic criteria based on conditions potentially related to cachexia, including undernutrition, to estimate and compare the prevalence and incidence of cancer cachexia in a US paediatric population across different tumour types and age groups.

## 2 Methods

### 2.1 Study Design and Study Population

This retrospective cohort study estimated annual paediatric cancer prevalence from 2017 to 2020, cancer incidence from 2017 to 2021, and cancer cachexia prevalence and incidence from 2017 to 2021. The Surveillance, Epidemiology, and End Results (SEER) database [24] was used to estimate the prevalence and incidence of cancer, while data from the Optum Enriched Oncology Electronic Health Records Database (Optum EHR) were used to estimate cancer cachexia prevalence and incidence. Patients in the Optum EHR database had to have: (1) an eligible cancer diagnosis during the cancer diagnosis capture period; and (2) either at least one body weight and height/length measure within the prespecified period, or at least one International Classification of Diseases, Tenth Revision, Clinical Modification (ICD‐10‐CM) cachexia‐related diagnosis code (Supplemental Table S1) between 12 months prior to cancer index date and the end of the study period. Patients (all aged < 18 years at cancer index date) were placed into age subgroups. For cancer estimates, these subgroups were < 1 year, 1 to < 5 years, 5 to < 10 years, and 10 to < 20 years. For cancer cachexia estimates, these were < 1 year, 1 to < 2 years, 2 to < 12 years, and 12 to < 18 years.

### 2.2 Definition of Cancer Exposure

Cancer was defined as having one inpatient (IP) or two outpatient (OP) diagnoses of the same cancer at least 30 days apart during the study period. If there were two OP diagnoses, the second diagnosis date was used as the index date. Information on cancer diagnosis was extracted from the SEER database [24]. The cancer diagnosis capture period was from 1 January 2017 until the most recent available record at the data cutoff on 31 December 2021, or when a patient became lost to follow‐up.

### 2.3 Definition of Cachexia Outcomes

Five different sets of candidate criteria were used to explore cachexia, which were defined based on conditions potentially related to cachexia, including undernutrition. Criteria 1–4 capture different severity thresholds for clinical measures of growth, based on standardised anthropometric measures for mild undernutrition or severe undernutrition. Two types of anthropometric indicators were included, z‐scores and body weight trajectory [20]. Criterion 5 included any ICD‐10‐CM cachexia‐related diagnosis code (Table 1). Body weight for length/height z‐scores were used for patients aged < 2 years, and body mass index z‐scores for patients aged ≥ 2 years, with thresholds based on published z‐score and percentage of normal expected weight gain cutoffs for ‘at least mild undernutrition’ and ‘severe undernutrition’ [20]. All z‐scores were computed using the SAS programme provided by the Centres for Disease Control and Prevention [25]. The sets of criteria were as follows:

**Table 1:** The five cachexia criteria used in this study.

Cachexia criterion	Definition
Criterion 1 (corresponding to at least mild undernutrition)	z‐score decline ≥ 1 with at least two eligible anthropometric measures, or z‐score ≤ –1 with a single measure
Criterion 2 (corresponding to severe undernutrition)	z‐score decline ≥ 3 with at least two eligible anthropometric measures, or z‐score ≤ –3 with a single measure
Criterion 3 (corresponding to at least mild undernutrition)	< 75% of normal expected weight gain or 5% loss of usual body weight with at least two eligible anthropometric measures, or z‐score ≤ –1 with a single measure
Criterion 4 (corresponding to severe undernutrition)	< 25% of normal expected weight gain or 10% loss of usual body weight with at least two eligible anthropometric measures, or z‐score ≤ –3 with a single measure
Criterion 5 (diagnosis‐based)	Any one IP or two OP diagnoses based on an ICD‐10‐CM cachexia‐related code for cachexia, undernutrition, abnormal weight loss, underweight, sarcopenia, or muscle wasting at least 30 days apart during the cachexia capture period

Abbreviations: ICD‐10‐CM, International Classification of Diseases, Tenth Revision, Clinical Modification; IP, inpatient; OP, outpatient.

Criterion 1 (corresponding to at least mild undernutrition): z‐score decline ≥ 1 with at least two eligible anthropometric measures, or z‐score –1 or lower with a single measure.

Criterion 2 (corresponding to severe undernutrition): z‐score decline ≥ 3 with at least two eligible anthropometric measures, or z‐score –3 or lower with a single measure.

Criterion 3 (corresponding to at least mild undernutrition): < 75% of normal expected weight gain (for patients aged < 2 years) or 5% loss of usual body weight (for patients aged ≥ 2 years) with at least two eligible anthropometric measures, or z‐score –1 or lower with a single measure.

Criterion 4 (corresponding to severe undernutrition): < 25% of normal expected weight gain (for patients aged < 2 years) or 10% loss of usual body weight (for patients aged ≥ 2 years) with at least two eligible anthropometric measures, or z‐score –3 or lower with a single measure.

Criterion 5 (diagnosis‐based): any one IP or two OP diagnoses based on an ICD‐10‐CM cachexia‐related code for cachexia, undernutrition, abnormal weight loss, underweight, sarcopenia, or muscle wasting (Supplemental Table S1) at least 30 days apart during the cachexia capture period. The cachexia index date was the first IP date or the second OP date.

The cachexia capture period was from 12 months prior to the cancer index date until 5 years after the cancer index date, or the most recent available data, or when a patient was lost to follow‐up. This ensured that cancer‐associated cachexia was adequately captured regardless of whether it occurred before cancer was diagnosed or after the diagnosis of cancer in patients living with cancer for an extended period of time [26].

The tumour type subgroups were lymphoid leukaemia (acute and chronic), acute myeloid leukaemia (AML), Hodgkin lymphoma, non‐Hodgkin lymphoma, brain and central nervous system (CNS) cancer, neuroblastoma and peripheral nerve tumours, nephroblastoma and other nonepithelial renal tumours, hepatic tumour, osteosarcoma, Ewing tumour and related bone sarcomas, rhabdomyosarcoma, germ cell and gonadal tumours, thyroid carcinoma, melanoma skin cancer and other cancers.

For criteria that used at least two eligible measures to define cachexia, the measures must have been taken within approximately 6 months (153–213 days) of each other, and the second measure must have been taken within the cachexia capture period. For criteria that used one eligible measure, that measure must have been taken during the cachexia capture period. The six potential scenarios for capturing eligible measures are shown in Supplemental Figure S1.

### 2.4 Prevalence of Cachexia

Prevalence was estimated by dividing the number of patients with estimated cancer cachexia in the specified period by the total number of patients with cancer in the specified period for each group defined by cancer type, age, and cachexia definition.

### 2.5 Incidence of Cachexia

Estimated incidence rates were calculated by dividing the number of estimated new cachexia patient cases in the specified period by the total number of patients with cancer at risk over the same period.

### 2.6 Ethics

This study was conducted in accordance with legal and regulatory requirements, as well as the research practices described in the Guidelines for Good Pharmacoepidemiology Practices issued by the International Society for Pharmacoepidemiology, European Medicines Agency European Network of Centres for Pharmacoepidemiology and Pharmacovigilance Guide on Methodological Standards in Pharmacoepidemiology, and the United States Food and Drug Administration (FDA) Guidance for Industry: Good Pharmacovigilance and Pharmacoepidemiologic Assessment, and FDA Guidance for Industry and FDA Staff: Best Practices for Conducting and Reporting of Pharmacoepidemiologic Safety Studies Using Electronic Healthcare Data Sets.

## 3 Results

### 3.1 Cancer Prevalence and Incidence

Based on the SEER database [24], between 2017 and 2020, the most prevalent types of cancer among patients aged < 20 years were lymphoid leukaemia, brain and CNS cancers, and other cancers. Similarly, from 2017 to 2021, incidence rates of lymphoid leukaemia, brain and CNS cancers, and other cancers were the highest (Supplemental Table S2), consistent with the expected distribution of cancer diagnoses in children [27]. These findings were broadly consistent irrespective of age group, although results were more variable in patients aged < 1 year.

### 3.2 Epidemiological Estimates of Cachexia

In total, 115,664,448 patients were identified in the Optum EHR database, and 70.4% (*n* = 81,389,934) were active in the database between 1 January 2016 and 31 December 2021. Of patients active in the database, 5.0% (*n* = 4,031,269) had an eligible malignant cancer diagnosis between 1 January 2017 and 31 December 2021, and 52.8% of these (*n* = 2,128,277) had database activity within 12 months before the cancer index date. In total, 26,855 patients were aged < 18 years at the first eligible cancer diagnosis and formed the study population (Table 2). Approximately half were male (50.1%), and the mean (standard deviation) age at first eligible cancer diagnosis was 10.1 (5.5) years. Patients aged ≥ 2 years constituted approximately 90% of the study population. The majority of patients were White (75.5%); Asian and Black/African American patients accounted for 2.9% and 7.9% of the population, respectively.

**Table 2:** Characteristics of the cancer patient population used to estimate cancer cachexia prevalence and incidence rate (Optum Enriched Oncology Electronic Health Records Database).

Characteristic	Population (*N* = 26,855)
Sex, *n* (%)	
Female	13,349 (49.7)
Male	13,466 (50.1)
Unknown	40 (0.2)
Age at first eligible cancer diagnosis, years	
Mean (SD)	10.1 (5.5)
Median (IQR)	11 (5–15)
Range	0‐17
First eligible cancer diagnosis by age group, *n* (%)	
< 1 year	1235 (4.6)
1–< 2 years	1556 (5.8)
2–< 12 years	10,790 (40.2)
12–< 18 years	13,274 (49.4)
Race, *n* (%)	
Asian	785 (2.9)
Black/African American	2113 (7.9)
White	20,272 (75.5)
Other/unknown	3685 (13.7)
Ethnicity, *n* (%)	
Hispanic	3391 (12.6)
Non‐Hispanic	20,310 (75.6)
Unknown	3154 (11.7)
Region, *n* (%)	
Midwest	10,451 (38.9)
Northeast	5186 (19.3)
South	6773 (25.2)
West	3380 (12.6)
Other/unknown	1065 (4.0)
Insurance types, *n* (%)^a^	
Commercial	17,861 (66.5)
Medicaid	6048 (22.5)
Medicare	172 (0.6)
Other payor type	657 (2.5)
Uninsured	1055 (3.9)
Unknown	1062 (4.0)

Abbreviations: IQR, interquartile range; SD, standard deviation.

^a^ As of the first date 12 months prior to the cancer index date.

Epidemiological estimates for cachexia varied considerably across the five different cachexia criteria. Generally, estimated prevalence and incidence rates were highest when using Criteria 1 and 3 (both equal to at least mild undernutrition), and lowest with Criterion 2 (equal to severe malnutrition).

### 3.3 Cachexia Prevalence

Based on data for 2021, in patients aged 2 to < 12 years (40.2% of the study population), according to Criterion 1, estimated cancer cachexia prevalence was lowest for thyroid carcinoma (29.4%) and highest for lymphoid leukaemia (60.3%); according to Criterion 2, it was lowest for germ gonadal tumours (5.0%) and highest for AML (30.0%); according to Criterion 3, it was lowest for melanoma skin cancer (6.5%) and highest for lymphoid leukaemia (50.4%); according to Criterion 4, it was lowest for germ gonadal tumours (4.0%) and highest for lymphoid leukaemia (32.5%); and according to Criterion 5, it was lowest for Hodgkin lymphoma (12.2%) and highest for Ewing tumour and related bone sarcomas (73.4%). In patients aged 12 to < 18 years (49.4% of the study population), estimated cachexia prevalence was lowest for thyroid carcinoma according to Criteria 1, 2, 4 and 5 (0.0%–7.2%) and for other cancers according to Criterion 3 (10.4%), and highest for AML according all five criteria (Table 3). In 2021, across all age groups, according to Criterion 1, estimated cachexia prevalence was lowest for thyroid carcinoma (10.4%) and highest for lymphoid leukaemia (51.0%) (Supplemental Table S3). According to Criteria 2 and 4, estimated cachexia prevalence was lowest for thyroid carcinoma (0.9%–3.5%) and highest for AML (33.3%). According to Criterion 3, estimated cachexia prevalence was lowest for melanoma (9.3%) and highest for lymphoid leukaemia (48.0%). According to Criterion 5, estimated cachexia prevalence was lowest for thyroid carcinoma (7.8%) and highest for Ewing tumour and related bone sarcomas (58.9%) (Supplemental Table S3). The population sample sizes used to calculate prevalence by tumour type and candidate cachexia criterion across individual years are shown in Supplemental Table S4. For the different tumour types, sample sizes were generally consistent with the relative prevalence and incidence of these cancers in paediatric patients in the SEER database, with lymphoid leukaemia, brain and CNS cancers, and other cancers being the most common (Supplemental Tables S2 and S3). However, the sample sizes often varied considerably. As shown in Supplemental Table S3, the 2021 prevalence of estimated cachexia using Criterion 5 was 7.8% among patients with thyroid carcinoma compared with 57.1% for AML, while the corresponding sample sizes were 115 and 14 patients, respectively.

**Table 3:** Cancer cachexia prevalence (%) in the United States by tumour type, cachexia criteria, and age group (Optum Enriched Oncology Electronic Health Records Database, 2021).

	Age group, years																			
	Criterion 1				Criterion 2				Criterion 3				Criterion 4				Criterion 5			
Cancer type, %	< 1	1 to < 2	2 to < 12	12 to < 18	< 1	1 to < 2	2 to < 12	12 to < 18	< 1	1 to < 2	2 to < 12	12 to < 18	< 1	1 to < 2	2 to < 12	12 to < 18	< 1	1 to < 2	2 to < 12	12 to < 18
Lymphoid leukaemia	0.0	58.3	60.3	33.6	0.0	8.3	14.9	5.4	0.0	0.0	50.4	44.6	0.0	0.0	32.5	28.2	0.0	33.3	29.4	31.2
Acute myeloid leukaemia	0.0	N/A	50.0	100.0	0.0	N/A	30.0	100.0	0.0	N/A	30.0	100.0	0.0	N/A	30.0	100.0	0.0	N/A	50.0	100.0
Hodgkin lymphoma	N/A	N/A	34.1	10.8	N/A	N/A	7.3	2.2	N/A	N/A	14.6	20.4	N/A	N/A	7.3	8.6	N/A	N/A	12.2	16.2
Non‐Hodgkin lymphoma	N/A	66.7	42.2	23.8	N/A	0.0	12.4	3.5	N/A	0.0	27.0	34.3	N/A	0.0	20.2	23.1	N/A	0.0	32.2	38.5
Brain and CNS cancer	0.0	30.0	37.6	19.3	0.0	10.0	10.5	3.1	0.0	0.0	19.9	23.8	0.0	0.0	11.8	11.8	50.0	40.0	28.1	18.7
Neuroblastoma and peripheral nerve tumours	16.7	47.1	51.0	25.4	0.0	17.6	12.2	6.5	0.0	0.0	22.9	27.2	0.0	0.0	14.5	16.9	16.7	29.4	28.3	26.0
Nephroblastoma and other non‐epithelial renal tumours	0.0	54.5	44.9	16.0	0.0	0.0	9.6	3.1	0.0	9.1	24.2	16.0	0.0	0.0	14.0	9.9	0.0	9.1	25.1	14.8
Hepatic tumours	50.0	55.6	58.4	25.7	50.0	11.1	19.9	1.4	0.0	0.0	24.7	29.7	0.0	0.0	15.7	25.7	50.0	22.2	47.6	36.5
Osteosarcoma	100.0	50.0	45.2	28.1	0.0	0.0	10.8	5.1	0.0	0.0	21.9	32.6	0.0	0.0	13.0	22.0	0.0	50.0	61.5	40.6
Ewing tumour and related bone sarcomas	N/A	N/A	37.8	33.1	N/A	N/A	9.6	6.3	N/A	N/A	19.7	36.8	N/A	N/A	11.2	23.8	N/A	100.0	73.4	47.3
Rhabdomyosarcoma	N/A	66.7	52.5	28.8	N/A	0.0	15.3	4.4	N/A	0.0	29.1	35.6	N/A	0.0	20.2	22.9	N/A	33.3	35.0	32.2
Germ cell and gonadal tumours	0.0	0.0	37.5	18.0	0.0	0.0	5.0	0.8	0.0	0.0	8.5	18.0	0.0	0.0	4.0	8.6	0.0	0.0	59.3	45.3
Thyroid carcinoma	N/A	0.0	29.4	7.2	N/A	0.0	5.9	0.0	N/A	0.0	11.8	15.5	N/A	0.0	5.9	3.1	N/A	0.0	17.6	6.2
Melanoma skin cancer	N/A	25.0	33.9	8.7	N/A	0.0	12.9	2.6	N/A	0.0	6.5	11.2	N/A	0.0	6.5	4.3	N/A	0.0	17.5	11.3
Other cancers	17.1	36.4	29.9	9.0	4.9	7.8	7.0	1.4	1.2	3.5	9.5	10.4	0.6	0.5	4.8	4.7	15.4	16.6	14.9	9.2
**Prevalence range across cancer types**	**0.0–100.0**	**0.0–66.7**	**29.4–60.3**	**7.2–100.0**	**0.0–50.0**	**0.0–17.6**	**5.0–30.0**	**0.0–100.0**	**0.0–1.2**	**0.0–9.1**	**6.5–50.4**	**10.4–100.0**	**0.0–0.6**	**0.0–0.5**	**4.0–32.5**	**3.1–100.0**	**0.0–50.0**	**0.0–100.0**	**12.2–73.4**	**6.2–100.0**

*Note:* Criterion 1: z‐score decline ≥ 1 with at least two eligible anthropometric measures, or z‐score ≤ –1 with a single measure; Criterion 2: z‐score decline ≥ 3 with at least two eligible anthropometric measures, or z‐score ≤ –3 with a single measure; Criterion 3: < 75% of normal expected weight gain or 5% loss of usual body weight with at least two eligible anthropometric measures, or z‐score ≤ –1 with a single measure; Criterion 4: < 25% of normal expected weight gain or 10% loss of usual body weight with at least two eligible anthropometric measures, or z‐score ≤ –3 with a single measure; Criterion 5: any one IP or two OP diagnoses based on an ICD‐10‐CM cachexia‐related code for cachexia, undernutrition, abnormal weight loss, underweight, sarcopenia, or muscle wasting at least 30 days apart during the cachexia capture period. For patients in the 2 to < 12 years and 12 to < 18 years age groups, blue shading indicates the highest prevalence by criterion; green shading indicates the lowest. Cancer cachexia prevalence ranges across different cancer types are shown in bold.

Abbreviations: CNS, central nervous system; ICD‐10‐CM, International Classification of Diseases, Tenth Revision, Clinical Modification; IP, inpatient; N/A, not available; OP, outpatient.

### 3.4 Cachexia Incidence

Estimated incidence rates also varied considerably by cachexia criterion (Table 4 and Supplemental Table S5). Again using 2021 as an example, estimated cachexia incidence rates ranged from 0 per 100,000 person years (PY) for AML to 32.5 per 100,000 PY for rhabdomyosarcoma based on Criterion 1; 0 per 100,000 PY for AML, germ gonadal tumours, or melanoma to 6.8 per 100,000 PY for hepatic tumours based on Criterion 2; 0 per 100,000 PY for AML to 35.7 per 100,000 PY for lymphoid leukaemia based on Criterion 3; 0 per 100,000 PY for AML, thyroid carcinoma, or melanoma to 19.7 per 100,000 PY for lymphoid leukaemia based on Criterion 4; and 0 per 100,000 PY for multiple different tumour types to 1.0 per 100,000 PY for osteosarcoma based on Criterion 5. The variation in PY at risk used to calculate estimated incidence rates by tumour type and cachexia criterion across individual years is shown in Supplemental Table S6.

**Table 4:** Cancer cachexia incidence rates per 100,000 person years in the United States by tumour type, cachexia criteria, and age group (Optum Enriched Oncology Electronic Health Records, 2021).

**Cancer type, incidence rates**	**Age group, years**																			
	**Criterion 1**				**Criterion 2**				**Criterion 3**				**Criterion 4**				**Criterion 5**			
	**< 1**	**1 to** < **2**	**2 to** < **12**	**12 to** < **18**	**< 1**	**1 to** < **2**	**2 to** < **12**	**12 to** < **18**	**< 1**	**1 to** < **2**	**2 to** < **12**	**12 to** < **18**	**< 1**	**1 to** < **2**	**2 to** < **12**	**12 to** < **18**	**< 1**	**1 to** < **2**	**2 to** < **12**	**12 to** < **18**
Lymphoid leukaemia	0.0	447.2	45.9	13.2	0.0	48.9	4.9	1.1	0.0	0.0	41.1	27.2	0.0	0.0	22.3	15.1	0.0	0.0	0.0	0.7
Acute myeloid leukaemia	0.0	N/A	0.0	N/A	0.0	N/A	0.0	N/A	0.0	N/A	0.0	N/A	0.0	N/A	0.0	N/A	0.0	N/A	0.0	N/A
Hodgkin lymphoma	N/A	N/A	21.2	5.7	N/A	N/A	7.5	0.0	N/A	N/A	8.0	18.8	N/A	N/A	0.0	5.5	N/A	N/A	0.0	0.0
Non‐Hodgkin lymphoma	N/A	0.0	33.4	8.5	N/A	0.0	7.7	2.2	N/A	0.0	26.4	23.5	N/A	0.0	16.7	11.3	N/A	0.0	0.0	0.0
Brain and CNS cancer	0.0	134.3	17.4	5.9	0.0	51.7	5.9	0.5	0.0	0.0	10.7	14.0	0.0	0.0	7.1	7.2	0.0	0.0	0.0	0.7
Neuroblastoma and peripheral nerve tumours	182.1	180.2	34.9	7.7	0.0	28.8	7.3	0.0	0.0	0.0	14.0	13.5	0.0	0.0	10.5	6.9	0.0	0.0	0.0	0.0
Nephroblastoma and other nonepithelial renal tumours	0.0	82.5	30.6	0.0	0.0	0.0	5.6	0.0	0.0	0.0	11.1	8.2	0.0	0.0	4.0	5.8	0.0	0.0	0.0	0.0
Hepatic tumour	0.0	156.4	40.5	5.3	0.0	35.7	8.7	0.0	0.0	0.0	9.2	11.2	0.0	0.0	8.3	10.5	0.0	0.0	0.0	0.0
Osteosarcoma	552.5	230.4	19.6	13.1	0.0	0.0	4.8	3.0	0.0	0.0	12.2	19.4	0.0	0.0	7.3	8.5	0.0	0.0	0.0	1.6
Ewing tumour and related bone sarcomas	N/A	N/A	17.0	18.9	N/A	N/A	5.0	4.0	N/A	N/A	11.3	25.5	N/A	N/A	6.8	10.0	N/A	N/A	0.0	0.0
Rhabdomyosarcoma	N/A	340.5	47.1	16.6	N/A	0.0	10.1	0.0	N/A	0.0	24.0	31.0	N/A	0.0	17.7	15.1	N/A	0.0	0.0	0.0
Germ cell and gonadal tumours	N/A	0.0	15.9	0.0	N/A	0.0	0.0	0.0	N/A	0.0	7.8	0.0	N/A	0.0	1.5	0.0	N/A	0.0	0.0	0.0
Thyroid carcinoma	N/A	0.0	25.0	3.8	N/A	0.0	18.3	0.0	N/A	0.0	19.6	8.3	N/A	0.0	0.0	0.0	N/A	0.0	0.0	0.0
Melanoma skin cancer	N/A	113.6	7.6	0.0	N/A	0.0	0.0	0.0	N/A	0.0	0.0	5.9	N/A	0.0	0.0	0.0	N/A	0.0	0.0	0.0
Other cancers	104.4	94.1	10.7	3.6	24.0	16.5	2.4	0.6	0.0	1.0	4.9	7.3	0.0	0.0	2.4	3.5	0.0	0.0	0.3	0.3
**Range of incidence rates across cancer types**	**0.0–552.5**	**0.0–447.2**	**0.0–47.1**	**0.0–18.9**	**0.0–24.0**	**0.0–51.7**	**0.0–18.3**	**0.0–4.0**	**N/A**	**0.0–1.0**	**0.0–41.1**	**0.0–31.0**	**N/A**	**N/A**	**0.0–22.3**	**0.0–15.1**	**N/A**	**N/A**	**0.0–0.3**	**0.0–1.6**

*Note:* Criterion 1: z‐score decline ≥ 1 with at least two eligible anthropometric measures, or z‐score ≤ –1 with a single measure; Criterion 2: z‐score decline ≥ 3 with at least two eligible anthropometric measures, or z‐score ≤ –3 with a single measure; Criterion 3: < 75% of normal expected weight gain or 5% loss of usual body weight with at least two eligible anthropometric measures, or z‐score ≤ –1 with a single measure; Criterion 4: < 25% of normal expected weight gain or 10% loss of usual body weight with at least two eligible anthropometric measures, or z‐score ≤ –3 with a single measure; Criterion 5: any one IP or 2 OP diagnoses based on an ICD‐10‐CM cachexia‐related code for cachexia, undernutrition, abnormal weight loss, underweight, sarcopenia, or muscle wasting at least 30 days apart during the cachexia capture period. For patients in the 2 to < 12 years and 12 to < 18 years age groups, blue shading indicates the highest incidence rates by criterion; green shading indicates the lowest. Cancer cachexia incidence rates across different cancer types are shown in bold.

Abbreviations: CNS, central nervous system; ICD‐10‐CM, International Classification of Diseases, Tenth Revision, Clinical Modification; IP, inpatient; N/A, not available; OP, outpatient.

The number of patients aged < 2 years was very small or zero for most cancer types, making it difficult to compare cachexia rates across age groups. However, the estimated prevalence and incidence of cachexia in children aged < 2 years appeared lower than those in children aged 2 to < 18 years for most cancer types.

## 4 Discussion

Unlike in adults, there are no formal criteria for defining cancer cachexia in children [17, 18]. This study was one of the first attempts to generate sets of candidate criteria to explore identification and prevalence and incidence estimates of cachexia in children. Our findings suggest that estimates for cachexia prevalence and incidence vary across different cancer types, years of study, age groups and sets of criteria used. This variability highlights the complexity of defining criteria specific to paediatric patients when identifying and treating children with cancer cachexia.

Childhood cachexia presents several unique challenges compared with adult cachexia [17]. Children experience fluctuations in body weight, height, and physical function due to various stages of growth, and body composition of fat and muscle vary with pubertal development, complicating the assessment and management of cachexia [28]. Age‐appropriate and validated QoL measures are difficult to identify and implement in paediatric populations as well. Although some validated measures are appropriate for older children, there is a paucity of tools for younger children and even less consensus on when to use patient‐reported outcomes versus caregiver‐reported measures [29, 30]. Additionally, there are significant differences in cancer types and the treatment modalities and regimens between children and adults [31]. Childhood cancers are often highly malignant, aggressive cancers that require multi‐modal treatment approaches with chemotherapy, radiation, and stem cell transplant, while children also have different cardiac, metabolic, and renal physiology that alters long‐term outcomes and toxicities compared with adults [31, 32]. The mechanisms and biomarkers of cachexia have begun to be examined in adults, but little research has been performed in children, and there is limited knowledge about its impact on long‐term survival [17].

Our results suggest that estimates of cachexia are generally lower in paediatric than adult cancer populations [7, 33], in particular in children aged < 2 years, where they are often zero and typically lower compared with older children. This may be due to the importance of maintaining appropriate growth in children such that cachexia or undernutrition tends to be treated or corrected sooner than in adults, particularly in younger children [34]. It is common practice to place enteric feeding tubes early in therapy [35, 36, 37], whereas the mechanisms for nutritional supplementation in adults are more highly variable. However, paediatric hospitals may or may not have dedicated dietician staffing, which may contribute to variability in nutritional support and affect growth outcomes [38]. Although anthropometric measurements are accepted as standard of care in children, there are rarely additional measures of body composition, such as measures of muscle mass [39]. The challenges of identifying body weight loss, undernutrition, and ultimately cachexia in paediatric patients have been discussed previously [40]. Better characterisation of these cachexia definitions and criteria will allow for more consistent study and ultimately therapeutic interventions for cachexia.

Our results show that the prevalence of cachexia using Criteria 1 and 3 (both equal to at least mild undernutrition) and Criterion 5 (diagnosis codes) were comparable. The similarity between estimates using these three sets of criteria may help reinforce the validity of the findings, although future studies may be required for formal validation. Diagnosis codes were previously shown to underestimate the number of patients receiving nutritional support and, by proxy, cachexia [41]; however, our findings indicated comparable levels of cachexia prevalence when using changes in growth parameters with a threshold of mild undernutrition versus cachexia‐related diagnosis codes. This could reflect that observed body weight‐based changes may be more consistently accompanied by a billed diagnosis related to cachexia. However, the inability to maintain appropriate caloric intake by mouth also meets criteria for malnutrition and could be an important criterion for a cachexia definition [41]. The use of more stringent criteria to estimate cachexia (i.e., Criteria 2 and 4 that both correspond to severe undernutrition) typically returned lower prevalence rates compared with Criteria 1, 3 and 5. Lower prevalence rates with these criteria would be expected, as fewer patients experience severe malnutrition. Future work is required to determine whether the use of more stringent criteria, such as 2 and 4, could help identify those patients who are at the greatest risk of the negative effects of cachexia and thus would benefit most from cachexia‐directed therapies. Alternatively, using more sensitive and less specific criteria based either on anthropometric measurements or diagnosis codes, such as 1, 3 or 5, which identify more patients, including some who may not yet have developed cachexia, could provide greater benefit in terms of reducing morbidity and mortality. However, it is important to note that while undernutrition may be related to cachexia, not all patients who meet the criteria for undernutrition, in particular mild undernutrition, have or will go on to develop cachexia, and thus using undernutrition as a measure of cachexia may overestimate the prevalence of cachexia among paediatric populations with cancer.

Despite the size of the Optum EHR database, and in line with the variability in prevalence and incidence rate of different cancers in paediatric patients based on SEER data, we observed considerable variability in sample sizes among different tumour types, which may have affected estimates. For example, AML showed consistently high annual cachexia prevalence estimates (Supplemental Table S3) but consistently small sample sizes (Supplemental Table S4). AML treatment is known to include highly intensive therapy with high rates of infectious, nutritional, and multi‐organ system complications [42, 43]. These data indicate the need for further study of the proposed cachexia criteria in the context of interventions specific to cancer diagnosis and treatment regimens, and also highlight the importance of using such criteria in prospective studies. Data generated from small sample sizes may lead to wide confidence intervals and limited statistical power, warranting cautious interpretation. However, retaining the granularity of including cancer type ensured that the data collected on cachexia epidemiology were as precise as possible. Tumour types for which accurate cachexia estimates could not be obtained due to their rarity in childhood (e.g., AML) and challenges with anthropometric assessments (i.e., inconsistent frequency of height/length and body weight assessments) suggest that additional research is needed to establish cachexia estimates.

As with other studies using real‐world data, data quality issues and biases may occur, leading to potential misclassification of cancer and cachexia based on diagnosis codes. For some cancer types and age subgroups, the number of patients was zero, leading to a considerable number of ‘not available’ rates. In addition, due to patient privacy issues, Optum EHR does not provide birth month or date information. As a result, patient ages can only be estimated in years, and age subgroups covering short time periods such as ‘aged < 1 year’ and ‘aged 1 to < 2 years’ cannot be more specific. Another potential limitation was that prevalence and incidence rates may have been underestimated because of low or inconsistent within‐person frequency of height/length and body weight assessments, with only diagnosis codes and limited anthropometric measurements available in the databases. Although this is one of the first studies to propose paediatric cachexia criteria that could potentially be used for screening or diagnosis, we did not have information on physical function, QoL, and body composition (including muscle mass) related to the study population. Adult cancer cachexia data suggest that these parameters could be components of a paediatric cancer cachexia definition, but further work in this area is needed. Without a consensus on how paediatric cancer cachexia should be defined, approaches such as the current one cannot conclusively document the existence of paediatric cancer cachexia but can only attempt to provide an estimate of its potential prevalence and incidence among paediatric patients, which may be biased by the proxies chosen. Nonetheless, this study has several strengths. Both SEER and Optum EHR are large databases enabling analyses by cancer type and age subgroup. Optum EHR uses enriched data with detailed cancer diagnosis information and provides high coverage of individuals with at least one body weight/height/length assessment, resulting in only around 2% of patients for most cancer types (ranging from 1.6% to 7.1%) being excluded from Criteria 1–4 analyses due to lack of anthropometric measurements (Supplemental Table S7). In addition, the use of real‐world data increases the applicability of the findings to real‐world cancer populations and reduces potential biases associated with clinical trial data. As a result, this work may increase awareness of cancer cachexia in children among healthcare professionals. It may also help lay the foundation for an alignment on consensus definitions for paediatric cancer cachexia, their validation in different datasets, and the development of algorithms for the diagnosis of cancer cachexia in paediatric patients.

In conclusion, this study provides one of the first attempts to develop candidate criteria to define paediatric cancer cachexia. These criteria and resulting data provide a framework to inform further research aimed at generating consensus criteria for the identification of childhood cancer cachexia. Such definitions will support the development of cancer cachexia treatments tailored to paediatric patients to improve symptoms and physical function while also mitigating the potential long‐term impacts of cachexia on growth and development in survivors of childhood cancer [17].

## Author Contributions

Daniel V. Runco, Adina R. Lemeshow, Stephen E. Schachterle, Ira Jacobs, Alexandra Palmer, and Xunming Sun were responsible for the study conceptualization. Guohai Zhou curated the data. Guohai Zhou, Stephen E. Schachterle, and Agnieszka Gajewska participated in the formal analysis. Adina R. Lemeshow contributed to the acquisition of funding and resources. Ira Jacobs and Xunming Sun contributed to the investigations. Daniel V. Runco, Guohai Zhou, Stephen E. Schachterle, Agnieszka Gajewska, Ira Jacobs and Xunming Sun were responsible for the methodology. Daniel V. Runco, Ira Jacobs, and Xunming Sun were responsible for project administration. Guohai Zhou and Agnieszka Gajewska were responsible for software. Daniel V. Runco, Adina R. Lemeshow, Stephen E. Schachterle, and Xunming Sun were involved in supervision of the work. Stephen E. Schachterle and Agnieszka Gajewska were responsible for validation. Daniel V. Runco contributed to the visualization of results. All authors contributed to writing, reviewing and editing of the article.

## Conflicts of Interest

Daniel V. Runco has provided consultancy for Pfizer and Day One Biopharmaceuticals Inc. At the time of the study, Xunming Sun, Guohai Zhou, Adina R. Lemeshow, Stephen E. Schachterle, Agnieszka Gajewska, Ira Jacobs, and Alexandra Palmer were employees of Pfizer and may have stock and/or stock options in Pfizer.

## Supporting information


**Figure S1:** Study design schematics for determining eligible anthropometric measures. (A) to (D): Four different scenarios for patients with two sets of body measures; (E) and (F): Two different scenarios for patients with one set of body measures.
**Table S1:** Cachexia‐related ICD‐10‐CM diagnosis codes used to determine cachexia. **Table S2:** Cancer prevalence (%) and incidence rates (per 100,000 PY) by tumor type and age group in the United States (SEER 2017–2021) [24].
**Table S3:** Annual cancer cachexia prevalence (%) in the United States by tumor type and cachexia criteria (Optum Enriched Oncology Electronic Health Records Database, 2017–2021).
**Table S4:** Sample sizes (n) for determining cancer cachexia prevalence by tumor type and cachexia criteria (Optum Electronic Health Records, 2017–2021).
**Table S5:** Annual cancer cachexia incidence rates per 100,000 person years in the United States by tumor type and cachexia criteria (Optum Enriched Oncology Electronic Health Records, 2017–2021).
**Table S6:** Person‐years at risk for determining cancer cachexia incidence rates by tumor type and cachexia criteria (Optum Electronic Health Records, 2017–2021).
**Table S7:** Percentage of patients from the Optum Electronic Health Records database who were excluded from analyses using Criteria 1–4 due to lack of height, length, and body mass index measurements by cancer type.

## Data Availability

Upon request, and subject to review, Pfizer will provide the data that support the findings of this study. Subject to certain criteria, conditions and exceptions, Pfizer may also provide access to the related individual de‐identified patient data. See https://www.pfizer.com/science/clinical‐trials/trial‐data‐and‐results for more information.
