# Supplementary material for: Defining Cachexia in Children With Cancer in the United States: Developing a Framework to Inform Clinical and Research Practice
Source: J Hum Nutr Diet. 2026 Aug 13;39(4):e70325. doi: 10.1111/jhn.70325 (PMC13470151; doi:10.1111/jhn.70325)
Supplement: Supplementary file 1 [file JHN-39-0-s001.docx]

**SUPPORTING INFORMATION**

**SUPPLEMENTAL FIGURE S1. Study design schematics for determining eligible anthropometric measures. (A) to (D): Four different scenarios for patients with two sets of body measures; (E) and (F): Two different scenarios for patients with one set of body measures**

**SUPPLEMENTAL TABLE S1. Cachexia-related ICD-10-CM diagnosis codes used to determine cachexia**

| **Code** | **Description** |
| --- | --- |
| R64 | Cachexia |
| E41 | Nutritional marasmus |
| E42 | Marasmic kwashiorkor |
| E43 | Unspecified severe protein-calorie malnutrition |
| E44.0 | Moderate protein-calorie malnutrition |
| E44.1 | Mild protein-calorie malnutrition |
| E46 | Unspecified protein-calorie malnutrition |
| R63.4 | Abnormal weight loss |
| M62.50 | Muscle wasting and atrophy, not elsewhere classified, unspecified site |
| M62.511 | Muscle wasting and atrophy, not elsewhere classified, right shoulder |
| M62.512 | Muscle wasting and atrophy, not elsewhere classified, left shoulder |
| M62.519 | Muscle wasting and atrophy, not elsewhere classified, unspecified shoulder |
| M62.521 | Muscle wasting and atrophy, not elsewhere classified, right upper arm |
| M62.522 | Muscle wasting and atrophy, not elsewhere classified, left upper arm |
| M62.529 | Muscle wasting and atrophy, not elsewhere classified, unspecified upper arm |
| M62.531 | Muscle wasting and atrophy, not elsewhere classified, right forearm |
| M62.532 | Muscle wasting and atrophy, not elsewhere classified, left forearm |
| M62.539 | Muscle wasting and atrophy, not elsewhere classified, unspecified forearm |
| M62.541 | Muscle wasting and atrophy, not elsewhere classified, right hand |
| M62.542 | Muscle wasting and atrophy, not elsewhere classified, left hand |
| M62.549 | Muscle wasting and atrophy, not elsewhere classified, unspecified hand |
| M62.551 | Muscle wasting and atrophy, not elsewhere classified, right thigh |
| M62.552 | Muscle wasting and atrophy, not elsewhere classified, left thigh |
| M62.559 | Muscle wasting and atrophy, not elsewhere classified, unspecified thigh |
| M62.561 | Muscle wasting and atrophy, not elsewhere classified, right lower leg |
| M62.562 | Muscle wasting and atrophy, not elsewhere classified, left lower leg |
| M62.569 | Muscle wasting and atrophy, not elsewhere classified, unspecified lower leg |
| M62.571 | Muscle wasting and atrophy, not elsewhere classified, right ankle and foot |
| M62.572 | Muscle wasting and atrophy, not elsewhere classified, left ankle and foot |
| M62.579 | Muscle wasting and atrophy, not elsewhere classified, unspecified ankle and foot |
| M62.58 | Muscle wasting and atrophy, not elsewhere classified, other site |
| M62.59 | Muscle wasting and atrophy, not elsewhere classified, multiple sites |
| M62.84 | Sarcopenia |
| R63.6 | Underweight |

**SUPPLEMENTAL TABLE S2. Cancer prevalence (%) and incidence rates (per 100,000 PY) by tumor type and age group in the United States (SEER 2017-2021)** [24]

| **Age group** | **<1 year** | | **1 to <5 years** | | **5 to <10 years** | | **10 to <20 years** | |
| --- | --- | --- | --- | --- | --- | --- | --- | --- |
| **Cancer type** | **Prevalence** | **Incidence rate** | **Prevalence** | **Incidence rate** | **Prevalence** | **Incidence rate** | **Prevalence** | **Incidence rate** |
| Lymphoid leukemia | 0.0006 | 2.1 | 0.0143 | 7.2 | 0.0221 | 3.4 | 0.0082 | 1.9 |
| Acute myeloid leukemia | 0.0003 | 1.5 | 0.0034 | 1.0 | 0.0017 | 0.4 | 0.0019 | 0.8 |
| Hodgkin lymphoma | 0.0000 | 0.0 | 0.0000 | 0.1 | 0.0009 | 0.3 | 0.0061 | 2.0 |
| Non-Hodgkin lymphoma | 0.0000 | 0.1 | 0.0011 | 1.0 | 0.0044 | 1.3 | 0.0055 | 1.7 |
| Brain and CNS cancer | 0.0020 | 3.3 | 0.0087 | 4.0 | 0.0119 | 3.4 | 0.0100 | 2.6 |
| Neuroblastoma and peripheral nerve tumors | 0.0003 | 0.5 | 0.0012 | 0.4 | 0.0003 | 0.1 | 0.0001 | 0.0 |
| Kidney and renal pelvis | 0.0003 | 1.0 | 0.0049 | 1.8 | 0.0036 | 0.5 | 0.0007 | 0.2 |
| Hepatic tumors | 0.0007 | 1.7 | 0.0031 | 0.7 | 0.0009 | 0.1 | 0.0003 | 0.1 |
| Osteosarcoma or Ewing tumor and related bone sarcomas | 0.0003 | 0.5 | 0.0005 | 0.2 | 0.0019 | 0.6 | 0.0045 | 1.4 |
| Rhabdomyosarcoma | 0.0017 | 3.3 | 0.0047 | 1.1 | 0.0032 | 0.7 | 0.0033 | 1.0 |
| Thyroid cancer | 0.0000 | 0.1 | 0.0001 | 0.0 | 0.0003 | 0.1 | 0.0048 | 2.0 |
| Germ cell and gonadal tumors | 0.0003 | 0.6 | 0.0009 | 0.2 | 0.0006 | 0.2 | 0.0034 | 1.5 |
| Melanoma skin cancer | 0.0000 | 0.0 | 0.0002 | 0.1 | 0.0003 | 0.1 | 0.0013 | 0.6 |
| Other cancers | 0.0063 | 10.6 | 0.0182 | 4.2 | 0.0091 | 1.7 | 0.0158 | 5.3 |

Prevalence (%) was estimated between 2017 and 2020 and incidence rates per 100,000 PYs were estimated between 2017 and 2021.

Abbreviations: CNS, central nervous system; PY, person years; SEER, Surveillance, Epidemiology, and End Results.

**SUPPLEMENTAL TABLE S3. Annual cancer cachexia prevalence (%) in the United States by tumor type and cachexia criteria (Optum Enriched Oncology Electronic Health Records Database, 2017-2021)**

|  | **Criterion 1** | | | | | **Criterion 2** | | | | | **Criterion 3** | | | | | **Criterion 4** | | | | | **Criterion 5** | | | | |
| --- | --- | --- | --- | --- | --- | --- | --- | --- | --- | --- | --- | --- | --- | --- | --- | --- | --- | --- | --- | --- | --- | --- | --- | --- | --- |
| **Cancer type, %** | **2017** | **2018** | **2019** | **2020** | **2021** | **2017** | **2018** | **2019** | **2020** | **2021** | **2017** | **2018** | **2019** | **2020** | **2021** | **2017** | **2018** | **2019** | **2020** | **2021** | **2017** | **2018** | **2019** | **2020** | **2021** |
| Lymphoid leukemia | 44.7 | 47.7 | 47.9 | 50.1 | 51.0 | 9.3 | 11.1 | 10.9 | 11.3 | 11.6 | 43.7 | 45.0 | 44.4 | 46.8 | 48.0 | 26.3 | 28.9 | 28.2 | 29.5 | 30.8 | 32.0 | 31.5 | 31.2 | 30.8 | 30.1 |
| Acute myeloid leukemia | 60.0 | 62.5 | 63.6 | 60.0 | 50.0 | 50.0 | 37.5 | 27.3 | 40.0 | 33.3 | 50.0 | 37.5 | 45.5 | 40.0 | 33.3 | 50.0 | 37.5 | 45.5 | 33.3 | 33.3 | 60.0 | 66.7 | 54.5 | 50.0 | 57.1 |
| Hodgkin lymphoma | 16.3 | 16.7 | 15.1 | 13.1 | 15.0 | 2.3 | 2.2 | 2.0 | 2.5 | 3.1 | 24.7 | 24.6 | 21.5 | 18.6 | 19.4 | 9.0 | 9.5 | 9.6 | 7.6 | 8.4 | 21.7 | 18.4 | 18.2 | 16.4 | 15.5 |
| Non-Hodgkin lymphoma | 25.0 | 27.0 | 27.1 | 27.4 | 31.4 | 1.7 | 6.6 | 6.3 | 5.6 | 6.8 | 30.0 | 31.3 | 32.0 | 30.5 | 31.1 | 16.9 | 20.4 | 21.9 | 21.5 | 21.7 | 39.8 | 34.2 | 35.1 | 35.6 | 35.6 |
| Brain and CNS cancer | 24.7 | 25.1 | 25.6 | 27.3 | 28.7 | 5.7 | 5.7 | 5.4 | 6.0 | 7.0 | 19.8 | 19.9 | 19.3 | 20.6 | 21.6 | 10.4 | 10.3 | 10.3 | 10.7 | 11.7 | 29.8 | 25.4 | 23.5 | 24.1 | 23.7 |
| Neuroblastoma and peripheral nerve tumors | 45.1 | 39.7 | 40.5 | 40.6 | 42.9 | 11.8 | 9.8 | 10.1 | 9.4 | 10.5 | 26.9 | 23.0 | 22.5 | 22.1 | 23.4 | 17.4 | 14.2 | 13.7 | 13.6 | 14.7 | 34.5 | 28.6 | 28.1 | 27.9 | 27.6 |
| Nephroblastoma and other nonepithelial renal tumors | 32.0 | 30.4 | 31.3 | 34.2 | 36.0 | 6.0 | 5.9 | 6.1 | 6.7 | 7.4 | 18.3 | 18.1 | 19.4 | 21.0 | 21.3 | 8.8 | 10.6 | 11.4 | 12.4 | 12.4 | 19.5 | 19.3 | 20.3 | 21.0 | 21.5 |
| Hepatic tumors | 47.5 | 47.5 | 45.6 | 44.1 | 48.6 | 12.9 | 13.9 | 12.9 | 12.3 | 14.3 | 31.9 | 30.7 | 28.7 | 25.3 | 25.1 | 19.0 | 20.5 | 20.3 | 17.4 | 17.9 | 52.0 | 45.5 | 43.8 | 44.2 | 43.4 |
| Osteosarcoma | 34.6 | 33.0 | 34.8 | 34.6 | 36.2 | 6.3 | 6.5 | 6.0 | 6.9 | 7.7 | 26.9 | 25.4 | 27.4 | 27.4 | 27.5 | 13.4 | 14.2 | 16.5 | 17.7 | 17.8 | 59.5 | 53.7 | 51.4 | 50.5 | 50.2 |
| Ewing tumor and related bone sarcomas | 34.2 | 33.6 | 34.7 | 34.1 | 35.1 | 6.1 | 6.5 | 5.9 | 7.0 | 7.7 | 26.5 | 27.1 | 28.9 | 28.6 | 29.3 | 13.1 | 15.1 | 17.1 | 18.8 | 18.3 | 68.2 | 64.8 | 61.1 | 60.5 | 58.9 |
| Rhabdomyosarcoma | 37.3 | 37.8 | 37.1 | 38.4 | 40.9 | 10.5 | 10.3 | 9.2 | 9.4 | 9.7 | 34.0 | 29.1 | 28.5 | 30.5 | 31.9 | 20.2 | 19.6 | 20.2 | 19.8 | 21.3 | 34.1 | 32.2 | 31.5 | 34.7 | 33.6 |
| Germ cell and gonadal tumors | 24.6 | 26.6 | 26.8 | 27.1 | 29.4 | 2.7 | 3.3 | 3.0 | 3.6 | 3.3 | 9.5 | 12.2 | 13.2 | 13.9 | 12.0 | 5.0 | 6.6 | 7.2 | 7.1 | 5.7 | 67.9 | 62.3 | 56.7 | 53.7 | 53.0 |
| Thyroid carcinoma | 7.5 | 7.1 | 11.5 | 12.5 | 10.4 | 1.5 | 2.0 | 0.8 | 0.0 | 0.9 | 22.1 | 14.0 | 13.9 | 17.4 | 14.8 | 9.1 | 5.1 | 5.7 | 7.4 | 3.5 | 8.6 | 6.1 | 7.4 | 8.9 | 7.8 |
| Melanoma skin cancer | 20.5 | 18.2 | 18.4 | 18.2 | 17.7 | 8.3 | 6.2 | 7.0 | 6.5 | 6.1 | 11.8 | 9.9 | 9.5 | 10.5 | 9.3 | 7.1 | 5.4 | 5.7 | 6.4 | 5.0 | 9.3 | 12.4 | 13.3 | 13.4 | 13.2 |
| Other cancers | 18.6 | 18.1 | 18.0 | 18.9 | 19.5 | 3.6 | 3.7 | 3.7 | 3.9 | 4.2 | 10.6 | 9.7 | 9.5 | 9.6 | 9.7 | 4.8 | 4.5 | 4.5 | 4.5 | 4.6 | 17.6 | 13.9 | 12.7 | 12.7 | 12.1 |

Criterion 1: z-score decline ≥1 with at least two eligible anthropometric measures, or z-score ≤ –1 with a single measure; Criterion 2: z-score decline ≥3 with at least two eligible anthropometric measures, or z-score ≤ –3 with a single measure; Criterion 3: <75% of normal expected weight gain or 5% loss of usual body weight with at least two eligible anthropometric measures, or z-score ≤ –1 with a single measure; Criterion 4: <25% of normal expected weight gain or 10% loss of usual body weight with at least two eligible anthropometric measures, or z-score ≤ –3 with a single measure; Criterion 5: any one IP or two OP diagnoses based on an ICD-10-CM cachexia-related code for cachexia, undernutrition, abnormal weight loss, underweight, sarcopenia, or muscle wasting at least 30 days apart during the cachexia capture period. Blue shading indicates the highest prevalence in each year by criterion; green shading indicates the lowest.

Abbreviations: CNS, central nervous system; ICD-10-CM, International Classification of Diseases, Tenth Revision, Clinical Modification; IP, inpatient; OP, outpatient.

**SUPPLEMENTAL TABLE S4. Sample sizes (n) for determining cancer cachexia prevalence by tumor type and cachexia criteria (Optum Electronic Health Records, 2017-2021)**

|  | **Criterion 1** | | | | | **Criterion 2** | | | | | **Criterion 3** | | | | | **Criterion 4** | | | | | **Criterion 5** | | | | |
| --- | --- | --- | --- | --- | --- | --- | --- | --- | --- | --- | --- | --- | --- | --- | --- | --- | --- | --- | --- | --- | --- | --- | --- | --- | --- |
| **Cancer type, n** | **Year** | | | | | | | | | | | | | | | | | | | | | | | | |
|  | **2017** | **2018** | **2019** | **2020** | **2021** | **2017** | **2018** | **2019** | **2020** | **2021** | **2017** | **2018** | **2019** | **2020** | **2021** | **2017** | **2018** | **2019** | **2020** | **2021** | **2017** | **2018** | **2019** | **2020** | **2021** |
| Lymphoid leukemia | 838 | 1129 | 1374 | 1532 | 1630 | 830 | 1122 | 1371 | 1529 | 1630 | 835 | 1121 | 1373 | 1531 | 1630 | 832 | 1120 | 1371 | 1530 | 1630 | 850 | 1144 | 1389 | 1537 | 1630 |
| Acute myeloid leukemia | 5 | 8 | 11 | 10 | 12 | 4 | 8 | 11 | 10 | 12 | 4 | 8 | 11 | 10 | 12 | 4 | 8 | 11 | 9 | 12 | 5 | 9 | 11 | 10 | 14 |
| Hodgkin lymphoma | 178 | 233 | 252 | 237 | 226 | 177 | 231 | 250 | 236 | 226 | 178 | 232 | 251 | 237 | 227 | 178 | 232 | 250 | 237 | 227 | 180 | 234 | 253 | 238 | 226 |
| Non-Hodgkin lymphoma | 120 | 196 | 225 | 234 | 236 | 116 | 196 | 223 | 233 | 235 | 120 | 198 | 225 | 233 | 235 | 118 | 196 | 224 | 233 | 235 | 128 | 199 | 228 | 236 | 236 |
| Brain and CNS cancers | 1314 | 1781 | 2063 | 2189 | 2266 | 1305 | 1767 | 2056 | 2181 | 2264 | 1311 | 1777 | 2060 | 2187 | 2264 | 1304 | 1772 | 2058 | 2182 | 2264 | 1320 | 1789 | 2074 | 2203 | 2267 |
| Neuroblastoma and peripheral nerve tumors | 288 | 436 | 529 | 613 | 665 | 279 | 430 | 525 | 608 | 665 | 283 | 435 | 529 | 612 | 666 | 282 | 431 | 527 | 610 | 665 | 296 | 437 | 534 | 617 | 664 |
| Nephroblastoma and other nonepithelial renal tumors | 253 | 359 | 428 | 476 | 517 | 250 | 358 | 428 | 475 | 517 | 251 | 359 | 428 | 476 | 517 | 250 | 359 | 428 | 475 | 517 | 256 | 362 | 434 | 477 | 517 |
| Hepatic tumors | 120 | 162 | 206 | 238 | 251 | 116 | 158 | 201 | 236 | 251 | 119 | 163 | 202 | 237 | 251 | 116 | 161 | 202 | 236 | 251 | 127 | 165 | 210 | 240 | 251 |
| Osteosarcoma | 445 | 546 | 584 | 583 | 586 | 441 | 542 | 581 | 583 | 585 | 442 | 543 | 584 | 583 | 585 | 439 | 543 | 583 | 583 | 585 | 454 | 547 | 590 | 584 | 586 |
| Ewing tumor and related bone sarcomas | 345 | 399 | 424 | 417 | 427 | 342 | 398 | 422 | 417 | 427 | 347 | 399 | 422 | 416 | 427 | 343 | 398 | 421 | 416 | 427 | 349 | 401 | 424 | 418 | 428 |
| Rhabdomyosarcoma | 212 | 286 | 348 | 385 | 413 | 209 | 281 | 347 | 383 | 413 | 212 | 282 | 347 | 384 | 414 | 208 | 281 | 347 | 384 | 414 | 220 | 289 | 349 | 389 | 414 |
| Germ cell and gonadal tumors | 264 | 308 | 332 | 339 | 333 | 262 | 303 | 332 | 338 | 332 | 263 | 304 | 333 | 339 | 332 | 262 | 303 | 332 | 339 | 332 | 268 | 305 | 335 | 339 | 332 |
| Thyroid carcinoma | 67 | 99 | 122 | 120 | 115 | 66 | 99 | 121 | 120 | 115 | 68 | 100 | 122 | 121 | 115 | 66 | 99 | 122 | 121 | 115 | 70 | 99 | 122 | 123 | 115 |
| Melanoma skin cancer | 88 | 132 | 158 | 170 | 181 | 84 | 130 | 158 | 170 | 181 | 85 | 131 | 158 | 172 | 182 | 84 | 130 | 158 | 171 | 181 | 86 | 137 | 158 | 172 | 182 |
| Other cancers | 5608 | 9717 | 13083 | 14580 | 15882 | 5491 | 9610 | 13010 | 14526 | 15868 | 5553 | 9661 | 13046 | 14550 | 15879 | 5493 | 9619 | 13019 | 14529 | 15870 | 5649 | 9751 | 13130 | 14630 | 15887 |

Criterion 1: z-score decline ≥1 with at least two eligible anthropometric measures, or z-score ≤ –1 with a single measure; Criterion 2: z-score decline ≥3 with at least two eligible anthropometric measures, or z-score ≤ –3 with a single measure; Criterion 3: <75% of normal expected weight gain or 5% loss of usual body weight with at least two eligible anthropometric measures, or z-score ≤ –1 with a single measure; Criterion 4: <25% of normal expected weight gain or 10% loss of usual body weight with at least two eligible anthropometric measures, or z-score ≤ –3 with a single measure; Criterion 5: any one IP or 2 OP diagnoses based on an ICD-10-CM cachexia-related code for cachexia, undernutrition, abnormal weight loss, underweight, sarcopenia, or muscle wasting at least 30 days apart during the cachexia capture period.

Abbreviations: CNS, central nervous system; ICD-10-CM, International Classification of Diseases, Tenth Revision, Clinical Modification; IP, inpatient; OP, outpatient.

**SUPPLEMENTAL TABLE S5. Annual cancer cachexia incidence rates per 100,000 person years in the United States by tumor type and cachexia criteria (Optum Enriched Oncology Electronic Health Records, 2017-2021)**

|  | **Criterion 1** | | | | | | **Criterion 2** | | | | | | **Criterion 3** | | | | | | **Criterion 4** | | | | | | **Criterion 5** | | | | |
| --- | --- | --- | --- | --- | --- | --- | --- | --- | --- | --- | --- | --- | --- | --- | --- | --- | --- | --- | --- | --- | --- | --- | --- | --- | --- | --- | --- | --- | --- |
| **Cancer type, incidence rates** | **Year** | | | | | | | | | | | | | | | | | | | | | | | | | | | | |
|  | **2017** | **2018** | **2019** | **2020** | **2021** | **2017** | | **2018** | **2019** | **2020** | **2021** | **2017** | | **2018** | **2019** | **2020** | **2021** | **2017** | | **2018** | **2019** | **2020** | **2021** | **2017** | | **2018** | **2019** | **2020** | **2021** |
| Lymphoid leukemia | 36.3 | 46.9 | 38.3 | 39.1 | 32.3 | 4.0 | | 7.5 | 4.1 | 5.1 | 3.6 | 42.8 | | 49.8 | 36.8 | 43.4 | 35.7 | 16.9 | | 27.1 | 16.4 | 20.6 | 19.7 | 0.2 | | 0.0 | 0.0 | 0.0 | 0.3 |
| Acute myeloid leukemia | 120.2 | 0.0 | 58.9 | 0.0 | 0.0 | 110.5 | | 0.0 | 0.0 | 0.0 | 0.0 | 120.6 | | 0.0 | 41.2 | 0.0 | 0.0 | 120.6 | | 0.0 | 41.2 | 0.0 | 0.0 | 0.0 | | 0.0 | 0.0 | 0.0 | 0.0 |
| Hodgkin lymphoma | 7.4 | 5.4 | 2.1 | 4.9 | 8.0 | 1.4 | | 0.8 | 0.0 | 2.2 | 1.4 | 12.0 | | 18.8 | 12.8 | 10.7 | 16.6 | 4.4 | | 7.7 | 4.9 | 3.5 | 4.4 | 0.6 | | 0.0 | 0.0 | 0.0 | 0.0 |
| Non-Hodgkin lymphoma | 11.4 | 22.7 | 10.6 | 10.4 | 16.8 | 1.8 | | 7.7 | 1.1 | 0.0 | 4.2 | 21.0 | | 31.6 | 21.0 | 14.4 | 24.5 | 7.0 | | 20.7 | 12.8 | 8.4 | 13.4 | 0.0 | | 0.0 | 1.6 | 0.0 | 0.0 |
| Brain and CNS cancer | 13.0 | 14.2 | 13.2 | 11.1 | 11.7 | 2.7 | | 2.4 | 1.2 | 2.2 | 3.4 | 11.0 | | 11.7 | 11.0 | 11.3 | 12.2 | 4.9 | | 6.0 | 5.5 | 4.0 | 7.1 | 0.3 | | 0.7 | 0.6 | 0.3 | 0.3 |
| Neuroblastoma and peripheral nerve tumors | 35.2 | 19.8 | 27.6 | 22.5 | 28.0 | 6.6 | | 3.2 | 6.5 | 1.9 | 5.4 | 15.6 | | 10.7 | 11.1 | 10.0 | 13.5 | 10.2 | | 6.8 | 7.3 | 5.6 | 9.2 | 0.0 | | 0.6 | 0.0 | 0.0 | 0.0 |
| Nephroblastoma and other nonepithelial renal tumors | 17.9 | 18.3 | 17.6 | 18.7 | 19.0 | 2.4 | | 2.8 | 3.0 | 2.4 | 3.6 | 9.9 | | 9.1 | 11.8 | 11.1 | 9.9 | 4.7 | | 8.2 | 6.4 | 5.7 | 4.5 | 0.0 | | 1.3 | 0.0 | 0.0 | 0.0 |
| Hepatic tumors | 43.9 | 36.5 | 29.4 | 15.0 | 30.7 | 7.3 | | 9.3 | 8.2 | 1.3 | 6.8 | 23.5 | | 12.8 | 13.4 | 7.6 | 9.3 | 11.0 | | 14.6 | 10.7 | 6.9 | 8.5 | 0.0 | | 0.0 | 0.0 | 0.0 | 0.0 |
| Osteosarcoma | 32.1 | 15.5 | 15.0 | 10.7 | 17.2 | 4.6 | | 3.3 | 0.9 | 2.5 | 3.8 | 34.3 | | 16.2 | 18.4 | 17.9 | 15.7 | 13.4 | | 10.5 | 11.6 | 11.7 | 7.9 | 1.9 | | 4.5 | 0.8 | 1.9 | 1.0 |
| Ewing tumor and related bone sarcomas | 40.0 | 17.7 | 11.8 | 9.0 | 18.0 | 5.8 | | 2.9 | 1.3 | 2.1 | 4.5 | 41.9 | | 21.3 | 20.1 | 16.6 | 18.2 | 16.2 | | 11.3 | 13.3 | 12.0 | 8.4 | 1.1 | | 8.3 | 1.5 | 1.6 | 0.0 |
| Rhabdomyosarcoma | 35.1 | 22.8 | 24.4 | 26.2 | 32.5 | 6.5 | | 5.4 | 3.1 | 5.4 | 4.8 | 30.3 | | 20.4 | 24.2 | 23.1 | 27.0 | 14.3 | | 14.6 | 15.5 | 11.4 | 16.3 | 1.5 | | 2.0 | 1.1 | 0.0 | 0.0 |
| Germ cell and gonadal tumors | 20.0 | 9.1 | 10.4 | 3.2 | 8.8 | 2.3 | | 1.5 | 0.7 | 1.6 | 0.0 | 8.6 | | 6.1 | 7.3 | 5.4 | 5.0 | 4.9 | | 4.7 | 4.5 | 0.8 | 0.9 | 1.2 | | 7.4 | 0.0 | 1.6 | 0.0 |
| Thyroid carcinoma | 1.5 | 3.3 | 7.3 | 2.5 | 6.6 | 0.0 | | 1.6 | 0.0 | 0.0 | 2.9 | 4.1 | | 10.6 | 8.3 | 23.3 | 10.2 | 1.6 | | 3.2 | 3.6 | 4.7 | 0.0 | 0.0 | | 0.0 | 0.0 | 0.0 | 0.0 |
| Melanoma skin cancer | 3.5 | 6.5 | 4.9 | 1.9 | 4.2 | 4.5 | | 1.4 | 2.9 | 0.0 | 0.0 | 3.6 | | 3.0 | 4.5 | 5.2 | 3.7 | 1.6 | | 1.4 | 1.4 | 1.7 | 0.0 | 0.0 | | 0.0 | 1.6 | 0.0 | 0.0 |
| Other cancers | 8.2 | 9.5 | 9.4 | 6.6 | 8.5 | 1.6 | | 1.9 | 1.7 | 1.4 | 1.8 | 4.6 | | 5.4 | 5.8 | 4.7 | 6.0 | 2.2 | | 2.6 | 2.8 | 1.9 | 2.9 | 0.1 | | 0.3 | 0.3 | 0.2 | 0.3 |

Criterion 1: z-score decline ≥1 with at least two eligible anthropometric measures, or z-score ≤ –1 with a single measure; Criterion 2: z-score decline ≥3 with at least two eligible anthropometric measures, or z-score ≤ –3 with a single measure; Criterion 3: <75% of normal expected weight gain or 5% loss of usual body weight with at least two eligible anthropometric measures, or z-score ≤ –1 with a single measure; Criterion 4: <25% of normal expected weight gain or 10% loss of usual body weight with at least two eligible anthropometric measures, or z-score ≤ –3 with a single measure; Criterion 5: any one IP or 2 OP diagnoses based on an ICD-10-CM cachexia-related code for cachexia, undernutrition, abnormal weight loss, underweight, sarcopenia, or muscle wasting at least 30 days apart during the cachexia capture period. Blue shading indicates the highest incidence rate in each year by criterion; green shading indicates the lowest.

Abbreviations: CNS, central nervous system; ICD-10-CM, International Classification of Diseases, Tenth Revision, Clinical Modification; IP, inpatient; OP, outpatient.

**SUPPLEMENTAL TABLE S6. Person-years at risk for determining cancer cachexia incidence rates by tumor type and cachexia criteria (Optum Electronic Health Records, 2017-2021)**

|  | **Criterion 1** | | | | | **Criterion 2** | | | | | **Criterion 3** | | | | | **Criterion 4** | | | | | **Criterion 5** | | | | |
| --- | --- | --- | --- | --- | --- | --- | --- | --- | --- | --- | --- | --- | --- | --- | --- | --- | --- | --- | --- | --- | --- | --- | --- | --- | --- |
| **Cancer type, person-years** | **2017** | **2018** | **2019** | **2020** | **2021** | **2017** | **2018** | **2019** | **2020** | **2021** | **2017** | **2018** | **2019** | **2020** | **2021** | **2017** | **2018** | **2019** | **2020** | **2021** | **2017** | **2018** | **2019** | **2020** | **2021** |
| Lymphoid leukemia | 396,406 | 300,936 | 300,399 | 286,263 | 275,684 | 804,596 | 535,129 | 534,230 | 512,141 | 494,959 | 376,590 | 311,220 | 314,793 | 303,972 | 294,088 | 572,343 | 413,343 | 425,586 | 402,516 | 391,605 | 620,724 | 415,969 | 405,925 | 395,616 | 392,244 |
| Acute myeloid leukemia | 832 | 766 | 1,699 | 1,464 | 1,537 | 905 | 3,012 | 2,798 | 2,196 | 2,267 | 829 | 1,916 | 2,429 | 2,196 | 2,267 | 829 | 1,916 | 2,429 | 2,196 | 2,267 | 810 | 1,836 | 2,020 | 1,830 | 1,887 |
| Hodgkin lymphoma | 163,123 | 111,413 | 95,780 | 81,663 | 62,131 | 208,642 | 130,746 | 108,538 | 90,729 | 71,025 | 124,596 | 95,995 | 85,947 | 74,704 | 60,391 | 182,354 | 117,225 | 101,214 | 85,211 | 67,658 | 166,876 | 111,468 | 90,023 | 78,168 | 62,168 |
| Non-Hodgkin lymphoma | 79,124 | 87,937 | 75,146 | 67,141 | 53,471 | 112,122 | 116,688 | 94,263 | 86,121 | 71,286 | 66,564 | 75,869 | 66,618 | 62,645 | 52,964 | 85,175 | 91,789 | 77,908 | 71,318 | 59,767 | 72,862 | 83,743 | 63,931 | 59,407 | 49,665 |
| Brain and CNS cancers | 783,053 | 696,754 | 653,250 | 593,012 | 555,432 | 1,079,129 | 883,546 | 828,969 | 762,943 | 725,164 | 781,513 | 734,720 | 706,495 | 647,544 | 615,871 | 956,540 | 843,279 | 784,832 | 722,943 | 690,010 | 882,781 | 746,281 | 681,319 | 622,043 | 593,687 |
| Neuroblastoma and peripheral nerve tumors | 116,552 | 146,663 | 126,996 | 133,464 | 132,248 | 213,007 | 221,196 | 198,862 | 205,551 | 205,308 | 166,405 | 187,148 | 170,997 | 179,108 | 177,968 | 195,668 | 206,703 | 190,992 | 196,909 | 196,202 | 167,675 | 178,004 | 162,225 | 165,852 | 167,260 |
| Nephroblastoma and other nonepithelial renal tumors | 162,122 | 125,427 | 119,431 | 117,880 | 115,517 | 250,632 | 175,825 | 168,005 | 167,357 | 165,122 | 202,393 | 154,464 | 143,791 | 143,593 | 141,951 | 232,990 | 171,699 | 156,386 | 156,640 | 156,146 | 216,350 | 149,069 | 143,812 | 140,608 | 139,631 |
| Hepatic tumors | 45,575 | 41,081 | 44,251 | 46,784 | 45,594 | 82,628 | 64,622 | 72,840 | 76,100 | 73,809 | 72,398 | 54,752 | 59,691 | 65,823 | 64,342 | 81,848 | 61,575 | 65,619 | 72,290 | 70,245 | 57,135 | 42,846 | 49,186 | 49,295 | 47,923 |
| Osteosarcoma | 168,448 | 167,514 | 152,865 | 139,849 | 127,775 | 261,347 | 245,352 | 218,024 | 200,801 | 185,189 | 160,359 | 178,774 | 163,134 | 156,318 | 146,584 | 217,216 | 208,900 | 190,342 | 178,931 | 165,083 | 161,219 | 133,795 | 118,829 | 107,677 | 98,371 |
| Ewing tumor and related bone sarcomas | 109,865 | 112,987 | 109,836 | 99,957 | 94,200 | 172,442 | 169,641 | 158,116 | 142,530 | 134,519 | 100,356 | 117,423 | 114,701 | 108,360 | 104,222 | 142,146 | 141,650 | 135,021 | 124,539 | 119,046 | 93,468 | 72,711 | 68,598 | 61,054 | 57,708 |
| Rhabdomyosarcoma | 102,493 | 91,918 | 86,195 | 87,786 | 83,159 | 169,102 | 130,602 | 129,616 | 129,150 | 124,781 | 112,371 | 98,258 | 94,925 | 99,387 | 96,462 | 140,057 | 116,246 | 109,366 | 114,090 | 110,642 | 135,655 | 99,529 | 94,938 | 95,068 | 91,644 |
| Germ cell and gonadal tumors | 100,163 | 98,485 | 105,732 | 93,316 | 79,708 | 131,973 | 132,629 | 138,619 | 123,985 | 109,497 | 104,944 | 114,071 | 123,412 | 110,224 | 99,625 | 121,345 | 126,874 | 132,934 | 119,030 | 106,682 | 82,409 | 67,657 | 70,876 | 61,698 | 51,037 |
| Thyroid carcinoma | 65,725 | 60,355 | 54,468 | 39,386 | 30,502 | 68,660 | 63,845 | 59,668 | 45,307 | 34,162 | 48,758 | 56,586 | 48,127 | 38,564 | 29,444 | 61,987 | 61,882 | 56,269 | 42,316 | 32,907 | 67,333 | 61,043 | 54,533 | 42,079 | 31,623 |
| Melanoma skin cancer | 56,789 | 61,704 | 61,054 | 51,803 | 48,059 | 67,143 | 70,294 | 69,663 | 59,360 | 55,587 | 56,231 | 66,740 | 66,188 | 57,807 | 54,358 | 63,336 | 69,054 | 70,262 | 59,891 | 56,317 | 64,696 | 68,933 | 61,717 | 55,445 | 52,138 |
| Other cancers | 3,850,501 | 4,758,399 | 4,747,804 | 4,483,731 | 4,135,814 | 4,903,350 | 5,600,319 | 5,577,347 | 5,289,246 | 4,937,243 | 4,231,116 | 5,145,419 | 5,205,889 | 4,984,924 | 4,670,713 | 4,720,011 | 5,516,035 | 5,512,134 | 5,253,899 | 4,918,888 | 4,412,553 | 5,104,811 | 5,087,705 | 4,829,304 | 4,517,782 |

Criterion 1: z-score decline ≥1 with at least two eligible anthropometric measures, or z-score ≤ –1 with a single measure; Criterion 2: z-score decline ≥3 with at least two eligible anthropometric measures, or z-score ≤ –3 with a single measure; Criterion 3: <75% of normal expected weight gain or 5% loss of usual body weight with at least two eligible anthropometric measures, or z-score ≤ –1 with a single measure; Criterion 4: <25% of normal expected weight gain or 10% loss of usual body weight with at least two eligible anthropometric measures, or z-score ≤ –3 with a single measure; Criterion 5: any one IP or two OP diagnoses based on an ICD-10-CM cachexia-related code for cachexia, undernutrition, abnormal weight loss, underweight, sarcopenia, or muscle wasting at least 30 days apart during the cachexia capture period.

Abbreviations: CNS, central nervous system; ICD-10-CM, International Classification of Diseases, Tenth Revision, Clinical Modification; IP, inpatient; OP, outpatient.

**SUPPLEMENTAL TABLE S7. Percentage of patients from the Optum Electronic Health Records database who were excluded from analyses using Criteria 1-4 due to lack of height, length, and body mass index measurements by cancer type**

| **Cancer type** | **Percentage of patients without height/weight/** **body mass index measurements** |
| --- | --- |
| Lymphoid leukemia | 1.6 |
| Acute myeloid leukemia | 4.5 |
| Hodgkin lymphoma | 1.7 |
| Non-Hodgkin lymphoma | 1.8 |
| Brain and CNS cancer | 2.2 |
| Neuroblastoma and peripheral nerve tumors | 1.7 |
| Nephroblastoma and other nonepithelial renal tumors | 1.9 |
| Hepatic tumors | 1.9 |
| Osteosarcoma | 2.2 |
| Ewing tumor and related bone sarcomas | 1.8 |
| Rhabdomyosarcoma | 1.7 |
| Germ cell and gonadal tumors | 1.7 |
| Thyroid carcinoma | 1.9 |
| Melanoma skin cancer | 5.0 |
| Other cancers | 7.1 |
